# P-1310. Cefiderocol Remains Active against US Clinical Isolates of Stenotrophomonas maltophilia Non-susceptible to Comparator Agents: Results from the SENTRY Antimicrobial Surveillance Program (2020–2024)

**DOI:** 10.1093/ofid/ofaf695.1498

**Published:** 2026-01-11

**Authors:** Frank Kung, Salvatore Lentini, Sean T Nguyen, Boudewijn L DeJonge, Joshua Maher, Rodrigo E Mendes, Hidenori Yamashiro, Yoshinori Yamano

**Affiliations:** Shionogi Inc, Basking Ridge, NewJersey; Shionogi Inc, Basking Ridge, NewJersey; Shionogi Inc., Florham Park, NJ; Shionogi Inc., Florham Park, NJ; Element Materials Technology/Jones Microbiology Institute, North Liberty, Iowa; Element Iowa City (JMI Laboratories), North Liberty, IA; Shionogi & Co., Ltd., Toyonaka, Osaka, Japan; Shionogi & Co., Ltd., Toyonaka, Osaka, Japan

## Abstract

**Background:**

*Stenotrophomonas maltophilia* is a non-fermenting, multidrug-resistant opportunistic pathogen with limited treatment options due to intrinsic resistance mechanisms, such as the production of the chromosomally encoded metallo-β-lactamase, L1, and the extended-spectrum β-lactamase, L2. The Infectious Disease Society of America listed cefiderocol (FDC), a siderophore conjugated cephalosporin with broad activity against Gram-negative bacteria, as a preferred agent in combination therapy for *S. maltophilia* infections. In this analysis, the *in vitro* activities of FDC and comparators, were evaluated against *S*. *maltophilia* clinical isolates, including non-susceptible (NS) subsets, collected in the US from 2020-2024 as part of the SENTRY Antimicrobial Surveillance Program.

**Table.**
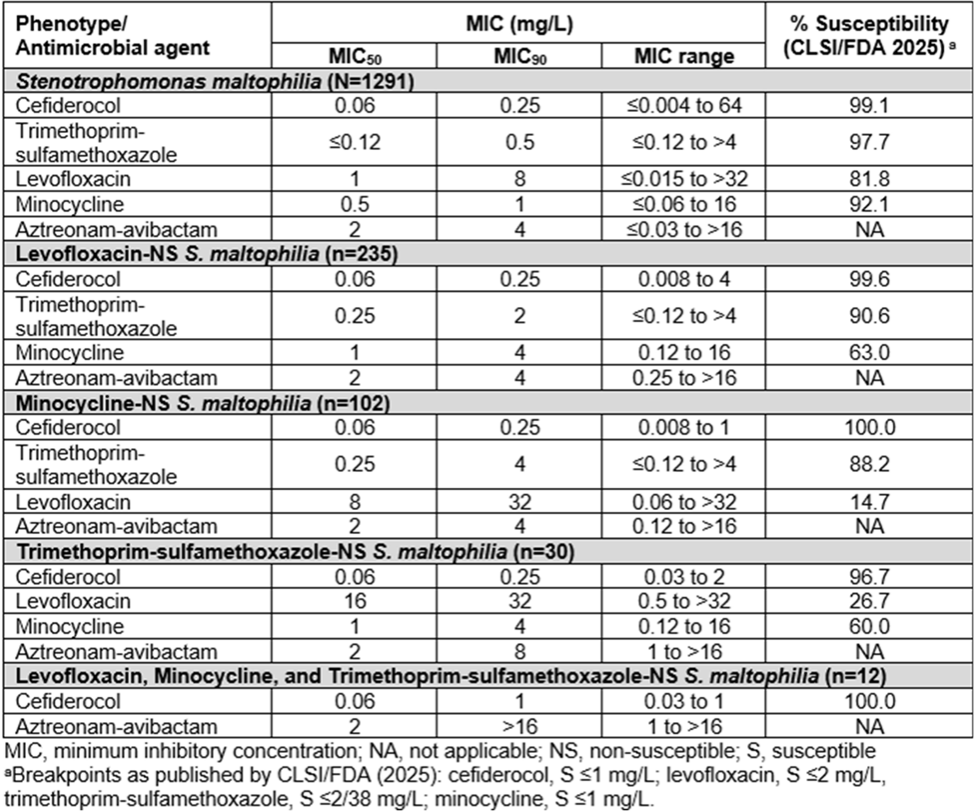
In Vitro Activity of Cefiderocol and Comparator Agents against US Isolates of Stenotrophomonas maltophilia (2020-2024)

**Methods:**

A total of 1,291 *S. maltophilia* isolates were consecutively collected from US patients between 2020-2024. Minimum inhibitory concentrations (MICs) were determined by using iron-depleted cation-adjusted Mueller-Hinton broth (ID-CAMHB) for FDC and CAMHB for comparators. MIC_50_, MIC_90_, and MIC ranges were calculated. Susceptibility was determined using CLSI/FDA breakpoints (2025; MIC ≤1 mg/L).

**Results:**

The most common infection types from which isolates were collected included pneumonia (n=943; 73.0%), skin/soft tissue (n=112; 8.7%), and blood stream infection (n=100; 7.7%). FDC was the most potent agent tested, with an MIC_50/90_ value of 0.06/0.25 mg/L, and 99.1% of the isolates being susceptible. MIC_50/90_ of aztreonam-avibactam was 2/4 mg/L (no interpretive breakpoint established). Susceptibilities to trimethoprim-sulfamethoxazole (SXT), levofloxacin (LVX), and minocycline (MIN) were 97.7%, 81.8%, and 92.1%, respectively. FDC maintained high susceptibility (≥96.7%) against isolates that were NS to LVX, SXT, or MIN and all of isolates NS to LVX, SXT, and MIN remained susceptible to FDC(Table).

**Conclusion:**

FDC showed potent *in vitro* activity against *S. maltophilia* collected in the US from 2020-2024, including against isolates NS to comparator antibiotics, suggesting that FDC is an important therapeutic option for infections caused by *S. maltophilia*.

**Disclosures:**

Frank Kung, PhD, Shionogi Inc: Employee Sean T. Nguyen, PharmD, Shionogi Inc: Employee Boudewijn L. DeJonge, PhD, Shionogi Inc.: Employee Rodrigo E. Mendes, PhD, GSK: Grant/Research Support|Shionogi & Co., Ltd.: Grant/Research Support|United States Food and Drug Administration: FDA Contract Number: 75F40123C00140 Hidenori Yamashiro, Shionogi HQ: Employee Yoshinori Yamano, PhD, Shionogi HQ: Employee

